# LncRNAs in pancreatic cancer

**DOI:** 10.18632/oncotarget.10545

**Published:** 2016-07-12

**Authors:** Xiaoyi Huang, Xiaosong Zhi, Yisha Gao, Na Ta, Hui Jiang, Jianming Zheng

**Affiliations:** ^1^ Department of Pathology, Changhai Hospital, Second Military Medical University, Shanghai, China; ^2^ Department of Cell Biology, Second Military Medical University, Shanghai, China

**Keywords:** pancreatic cancer, lncRNA, pathogen, mechanism, clinical

## Abstract

Pancreatic cancer (PC) is one of the most common causes of cancer-related death. The underlying mechanism of PC is not completely understood at present. Studies in recent years have demonstrated that long non-coding RNAs (lncRNAs) have multiple biological functions in cell growth, differentiation and proliferation. Notably, expressions of some lncRNAs undergo significant changes in the initiation and progression of cancers. In addition, lncRNAs are reported to be involved in various steps of PC development and have a potential value in the diagnosis, treatment and prognostic prediction of PC. In this review, we highlight recent evidence related to the molecular mechanism of lncRNAs in growth, survival, invasion, metastasis, angiogenesis and apoptosis of PC cells, and discuss the potential clinical application of lncRNAs to the diagnosis, treatment and prognostic prediction of PC.

## INTRODUCTION

Pancreatic cancer (PC) is a malignant neoplasm with a high mortality rate. The five-year survival rate of PC remains very low despite the use of comprehensive therapies [1]. Given the concealed location, PC is hardly discovered until serious clinical symptoms and signs are present, making early detection and diagnosis of PC a clinical challenge. Additionally, the therapeutic efficacy of PC is still far from satisfaction despite aggressive treatment [2, 3]. Although previous studies have identified numerous susceptibility loci for PC [4, 5, 6], the mechanism underlying transcriptome regulation is not well elucidated in PC. Inspiringly, recent studies have demonstrated that long non-coding RNAs (lncRNAs) is a critical factor in the pathogenesis of PC. More importantly, some lncRNAs may become potential biomarkers and/or drug targets for PC.

The Human Genome Project has revealed that protein-coding genes represent less than 2% of the total genome sequence [7], and the remaining greater portion of human genomes are regarded as “junk DNAs”, for they do not encode any protein. In addition, most of them are intron DNAs in animals, termed as non-coding DNAs (ncDNAs) [8, 9, 10]. Generally, ncRNAs are divided into two categories according to their length: small ncRNAs (<200bps) and long ncRNAs (>200bps) [11]. Although there is little knowledge about lncRNAs at present, their potential implications in gene transcriptomics have aroused great interest and attention in the most recent years [12].

Although lncRNAs cannot encode any functional protein, they are involved in diverse biological processes, playing essential roles in maintaining cell growth, differentiation and proliferation [13-15]. Notably, increasing data suggests that lncRNAs may play an irreplaceable role in the progression of autoimmune diseases and cancers [16, 17]. For instance, PVT1 serves as a promoting factor in gastric carcinogenesis and may become a candidate prognostic biomarker for gastric cancer [15]. Urinary lncRNA PCA3 can improve early detection of prostate cancer in prostate biopsy [18]. LncRNA metastasis-associated lung adenocarcinoma transcript 1 (MALAT-1) can be used to predict whether patients with non-small cell lung cancer (NSCLC) are at high risk of developing metastasis [19]. Although genomic analyses have discovered growing numbers of lncRNAs [20], most of them lack valid research and mechanisms of lncRNAs in tumorigenesis including PC remain largely unknown. The present review concentrates on the known mechanisms of lncRNAs in PC and discusses their potential clinical utility in PC.

## CLASSIFICATION AND FUNCTIONS OF LNCRNAs

lncRNAs can be classified as five categories according to their genomic location: (1) stand-alone lncRNAs, whose locations are away from protein-coding genes; (2) natural antisense transcripts, which are located on the opposite strand of annotated transcription units; (3) pseudogenes, whose transcripts have lost the protein coding potential since mutations; (4) long intronic ncRNAs, whose lncRNAs transcripts are from introns of annotated genes; and (5) divergent transcripts, promoter-associated transcripts and enhancer RNAs, whose lncRNAs arise from both sense and antisense directions of transcription start areas [21]. Although most lncRNA need to be verified functionally, it is of great importance to summarize current knowledge about lncRNA functions. According to previous studies, the proposed functions associated with lncRNA can be classified as follows: (1) recruiting and interacting with protein: lncRNA HOX transcript antisense RNA (HOTAIR) combines with Polycomb Repressive Complex 2 (PRC2) to regulate the transcription of homeobox D cluster (HOXD) gene [22]; (2) acting as a decoy: lncRNA termed P21 Associated NcRNA DNA damage activated (PANDA) acts as an decoy for nuclear transcription factor Y subunit A (NF-YA) to limit expression of pro-apoptotic genes [23]; (3) acting as a co-regulator or a co-repressor: a lncRNA termed Steroid receptor RNA activator (SRA) serves as a co-activator for a number of nuclear steroid receptors [24, 25]; (4) interacting with miRNA: lncRNA HOTAIR serves as a miRNA sponge of miR-331-3p to regulate HER2 expression in gastric cancer [26]; and (5) acting as host genes for miRNA: H19 serves as the reservoir of miR-675 which suppresses growth and insulin-like growth factor 1 receptor gene [27].

## ROLES OF LNCRNAs IN THE PATHOGENESIS OF PC

Mounting evidence has revealed that lncRNAs play important roles in the pathogenesis of various cancers including PC [28]. Give the diverse functions of lncRNAs in cancer biology, they may be used as potential biomarkers for early clinical diagnosis, treatment and prognostic predictions of cancers [29]. Although increasing studies have focused on lncRNAs and cancer, the underlying mechanisms of lncRNA in cancer and other diseases remain largely unknown. However, some useful clues have suggested the effect and diverse biological functions of lncRNA in cancers. Herein we summarize the action mechanisms of lncRNAs in PC and their potential clinical values. The functions of several important lncRNAs in PC and their potential clinical usages are shown in Table 1.

**Table 1:** The characteristics and functions of lncRNAs related to PC

LncRNA	Genomic location	RNA description	Expression change in PC cell or patients samples	Relevant targets in PC	Pro-oncogenic functions in cancers
HOTAIR [22, 33, 34, 35, 36]	12q13.13	HOX transcript antisense RNA	↑	PRC2, GDF15	Related to cancer cell invasion, proliferation, progression and invasion.
HOTTIP [38, 95]	7q15.2	HOXA transcript at the distal tip	↑	AURKA, WDR5, HOXA10, HOXB2, HOXA11, HOXA9, HOXA1, HOXA13	Promote cancer cell proliferation; inhibit cell apoptosis, increased migration.
MALAT-1 [19, 50, 53]	11q13.1	Metastasis-associated lung adenocarcinoma transcript-1	↑	Sox2, E-cadherin, N-cadherin, vimentin, VEGF	Regulated cell cycle, growth, migration and invasion
ENST00000480739 [43]	12q13.3	lncRNA ENST00000480739	↓	OS-9, HIF-1	Regulate invasion and migration.
AFAP1-AS1 [28, 81]	4p16.1	Actin filament associated protein 1 antisense RNA	↑	unknown	Regulate Cell proliferation, migration and invasion.
BC008363 [82]	5q21.2	lncRNA BC008363	↓	unknown	-
H19 [46, 47, 48, 49, 50, 51]	11q15.5	lncRNA H19	↑	Let-7, HMGA2	-
PVT1 [15, 85]	8q24.21	Plasmacytoma variant translocation 1	↑	unknown	-
GAS5 [39]	1q25.1	Growth arrest-specific 5	↓	CDK6	Inhibit cell proliferation.
AF339813 [64]	13q32	lncRNA AF339813	↑	NUF2, CDK1, CDK4/CDK6	Apoptosis, regulate cell cycle

## LNCRNAs IN CANCER CELL GROWTH AND SURVIVAL

Generally, the expression of lncRNAs undergoes changes in cancer cells, thus affecting lncRNA-miRNA and protein interactions [27, 30]. Some recent studies have reported their findings about the mechanism of lncRNAs in carcinogenesis and cancer progression in some cancer types including PC. Li et al reported that lncRNA NUTF2P3-001 induced by hypoxia promoted cell proliferation in Panc-1 and BXPC-3 cell lines, which was accompanied with increased KRAS expression [31]. Gao et al found that the lncRNA regulator of reprogramming (ROR) served as a competitive endogenous RNA (ceRNA) to decrease Nanog gene expression by sponging miR-145 in order to promote cell proliferation and carcinogenesis in both BxPC-3 and Capan-1 cell lines [32].

HOTAIR as a HOX antisense intergenic RNA can physically combine with PRC2, resulting in histone methylation and HOXD locus transcription silencing [22]. In PC, HOTAIR was up-regulated and functioned as an “onco-lncRNA” to promote cell proliferation, regulate cell cycle progression, and inhibit cancer cell apoptosis [33]. In vitro experiments suggested that HOTAIR regulated cell cycle progression, proliferation and apoptosis through PRC2 in Panc-1and L3.6pl cell lines, either dependently or independently. In addition, when the enhancer of zeste homolog2 (EZH2), a functional subunit of PRC2, was knocked down, cell proliferation was decreased and apoptosis was increased due to the presence of growth differentiation factor 15 (GDF15). In other cancer types such as renal cancer, colorectal adenocarcinoma and prostate cancer, HOTAIR was down-regulated by miR-141 [34], but whether this mechanism of HOTAIR regulation exists in PC is unknown. Collectively, as is the case with other cancers [35, 36], HOTAIR facilitates cell proliferation, regulates cell cycle and inhibits cell apoptosis through PRC2 and GDF15 mRNA in PC.

Another lncRNA related to HOX gene is the lncRNA termed HOXA transcript at the distal tip (HOTTIP). HOTTIP can promote H3 lysine 4 trimethylation by binding with WD repeat containing protein 5(WDR5) [37]. Similar to HOTAIR, HOTTIP is over-expressed in PC cell lines. The expression level of 757 genes was decreased and that of 514 genes was increased when HOTTIP was knocked down. Among these down-regulated genes, Aurora kinase A (AURKA), acting as a cell growth regulator, could maintain Panc-1 cell growth, inhibit apoptosis and promote migration, and these regulatory effects were independent of WDR5. However, siHOTTIP decreased the proportion of cells in S phase and increased of the proportion of cells in G_2_/M phase, while siAURKA decreased the percentage of cells in G0/G1 phase and increased the percentage of cells in S/G2 phase in Panc-1 PC cell line. This difference indicates that the mechanism of HOTTIP in promoting Panc-1 pancreatic cancer cell proliferation and survival through AURKA is more complex than we imagine. In hepatocellular carcinoma (HCC), the expression of HOXA locus gene was co-regulated by HOTTIP and WDR5/ mixed lineage leukemia 1 (MLL1) complex [38]. Unlike liver cancer cells, HOXA13 is not the downstream target of HOTTIP, but other HOX genes, such as HOXA10, HOXB2, HOXA9 and HOXA1 are increased by HOTTIP.

Unlike HOTTIP and HOTAIR, lncRNA GAS5 exhibited antiproliferative activity in PC cells. The expression level of GAS5 was significantly decreased in PC, indicating that it could promote cell proliferation via cyclin-dependent kinase 6 (CDK6) [39]. Nevertheless, how lncRNAs participate in PC onset remains largely unknown.

## LNCRNAs IN INVASION AND METASTASIS

Invasion and metastasis are important biological behaviors of all malignancies, making surgical resection and other radical treatments impossible. In PC, nerve invasion is an aggressive behavior indicator and related to poor prognosis in PC patients [40]. Besides, PC metastasis often occurs in the liver, lung and other organs [41, 42]. Due to lack of effective methods for early detection and diagnosis of PC, most PC patients have lost the best therapeutic moment at the time of diagnosis because of invasion and metastasis. Increasing evidence has indicated that lncRNAs regulate cancer invasion and metastasis, and therefore the mechanism underlying the role of lncRNAs in PC should be addressed, knowing that some lncRNAs may serve as potential indicators for PC invasion and metastasis.

In 2013, Kim et al [33] conducted a Boyden chamber assay in MiaPaCa2 and Panc28 cell lines and found that up-regulation of HOTAIR could enhance cell invasion. Similarly, when HOTTIP was knocked down in Panc1, migration was decreased [37]. Sun and colleagues [43] found that the lncRNA ENST00000480739 expression level was significantly decreased in PC, compared with that in the corresponding adjacent non-tumor tissue. Additionally, lncRNA ENST00000480739 expression level was negatively correlated with the prognosis of PC patients. Further analysis revealed that the mechanism underlying this regulatory role of lncRNA in cancer metastasis may be through up-regulating osteosarcoma amplified-9 (OS-9), a protein interacting with hypoxia-inducible factor 1 (HIF-1) and HIF-1 alpha prolyl hydroxylases [44]. Over-expression of HIF-1 and HIF-1 in PC tissue was reported to play a critical role in cell adaption to hypoxia and PC invasion and metastasis [45]. Similar to other HIF-1 inhibitors, new candidates targeting lncRNA ENST00000480739/OS-9/HIF-1 pathway could be promising drugs for PC. Another study [31] revealed that HIF-1α up-regulated lncRNA-NUTF2P3-001 under hypoxia, which could promote cell invasion in Panc-1 cell lines.

Another example is H19. H19, also known as imprinted maternally expressed transcript, is only expressed from maternally-inherited chromosome and serves as a tumor suppressor in several types of cancer [46]. Located on chromosome 11q15.5 with a length about 2.3kb, H19 is an important epigenomic regulator that controls genomic imprinting during development and growth [47]. Recent studies [48] reported that H19 promoted tumor metastasis via miR-675 and accelerated the epithelial-to-mesenchymal transition (EMT) progression. In liver cancer, H19 activated miR-200 pathway by promoting histone acetylation so as to suppress HCC metastasis and accelerate EMT [49]. Unlike the cases with HCC, H19 promoted gastric cancer cell proliferation, migration, invasion and metastasis through binding with isthmin1 protein. Besides, H19 can enhance carcinogenesis and metastasis in gastric cancer via miR-675 and calneuron 1(CALN1) [50]. H19 expression was up-regulated in the PC cancer tissue compared with that in the adjacent non-cancer tissue. The expression level of H19 in primary tumors with metastasis was higher than that in tumors without metastasis [51]. Furthermore, Ma et al [51] demonstrated that H19 promoted cell invasion and metastasis partially by antagonizing let-7(a miRNA), which can increase EMT mediated by High Mobility Group-A2 (HMGA2).

LncRNA MALAT-1 was first discovered in non-small cell lung cancer (NSCLC) [19]. What is more, MALAT-1 was also over-expressed in other types of cancer [52].MALAT-1 may serve as an oncogenic long noncoding RNA in PC by promoting EMT and increasing the expression of cancer stem cell markers [53]. Jiao and colleagues [54] found that MALAT-1 was over-expressed in cancer stem cells and could increase the percentage of PC stem cells through maintaining the self-renewing capacity, increasing resistance to anticancer drugs and promoting tumor angiogenesis in PC cell lines. During the process of EMT, the level of E-cadherin decreases while the expression of vimentin and N-cadherin increases. They also found that MALAT-1 expression was suppressed in PC cell lines, while the expression of E-cadherin was increased and the expression of N-cadherin and vimentin was decreased. Besides, the expression of Snail and Slug, two EMT-related transcriptional factors, were also down-regulated. The xenograft experiment in nude mice [53] revealed that MALAT-1 enhanced stemness of PC cells. Further analysis indicated that MALAT-1 maintained stemness of PC cells by Sox2. A recent study [55] found that MALAT-1 could recruit enhancer of zeste homolog 2 (EZH2) to the E-cadhherin promoter to promote cell migration and invasion in PC cell lines. Therefore, lncRNA MALAT-1 may promote the invasiveness and metastasis of PC.

Above all, lncRNAs can affect PC invasion and metastasis through lncRNA-miRNA interaction and/or lncRNA-protein interaction. However, the underlying mechanism of lncRNAs in invasion and metastasis of PC needs to be explored further. Since primary PC with invasion and metastasis is correlated with an advanced clinical stage and poor prognosis, it is urgent to develop a practicable therapy for patients with distant metastasis. Due to the key role of lncRNAs in PC, they can serve as potential prognostic biomarkers and therapeutic targets for PC, especially in advanced PC.

## LNCRNAs IN ANGIOGENESIS

Vasculature is essential for tumors to obtain nutrients and oxygen and evacuate metabolic wastes. Thus, angiogenesis is of great importance in the pathogenesis of cancer to maintain tumor expansion in a short time, especially in advanced stage cancer. During tumor progression, angiogenesis is activated by the net of cell signal pathways, which experience dramatic changes in carcinogenesis, and gene expression as well. In other words, the “angiogenic switch” including proangiogenic factors turns on during tumor progression. Vascular endothelial growth factor (VEGF) is a widely accepted proangiogenic factor. It is activated inappropriately from early to advanced stages in carcinogenesis [56]. Besides, VEGF gene is up-regulated when it is stimulate by hypoxia or oncogene signaling [57]. Similarly, other proangiogenic genes, such as fibroblast growth factor (FGF) and matrix metalloproteinase-9 (MMP-9), are also up-regulated in tumorigenesis [58]. In PC, thrombospondin-1(TSP-1) interacts with somatostatin receptor subtype 2 (sst2) to inhibit neoangiogenesis by directly suppressing VEGF [59].

lncRNAs have been evidenced to function diverse regulatory mechanisms in the angiogenic process. αHIF, an antisense RNA, can negatively adjust angiogenesis in cancer by regulating the expression of hypoxia-inducible factorα (HIF1α) [60]. Recently, a novel lncRNA termed associated with microvascular invasion in HCC (MVIH), was found to be up-regulated in HCC [61]. lncRNA MVIH is related to microvascular invasion, higher tumor node metastasis stage, decreased recurrence-free survival (RFS) and overall survival (OS). Importantly, lncRNA MVIH activates angiogenesis by inhibiting the secretion of phosphoglycerate kinase 1 (PGK1) [61, 62]. Additionally, lncRNA MVIH expression level is in relation to microvessel density (MVD) in clinical samples and inversely correlates with serum level of PGK1. In AsPC-1 cell lines, MALAT-1 can promote angiogenesis through increasing human umbilical vein endothelial cell (HUVEC) migration, tube length, the number of branch points and tube complexity [53]. Besides, MALAT-1 can affect the expression level of VEGF in AsPC-1 cells. However, the mechanism of lncRNAs in regulating angiogenesis in PC has not been well documented, although angiogenesis is of great importance in the initiation and progression of PC.

## LNCRNAs IN CELL APOPTOSIS

In the initiation and progression of cancer, cancer cells can attenuate apoptosis and therefore become drug resistant, which is a major barrier to effective cancer treatment [63]. Some recent studies reported that lncRNAs played an important role in cell apoptosis, including PC cell lines. Kim et al [33] demonstrated that knockdown of HOTAIR could induce the apoptosis in Panc 1 and L3.6pL cells. Also, knockdown of HOTTIP by RNA interference in Panc1cells lines led to cell apoptosis [37]. Bioinformatics analysis by Hu et al [64-66] revealed that lncRNA AF339813 was up-regulated by NUF2 (NUF2, Ndc80 kinetochore complex component), also known as cell division associated 1 (CDCA1), a component of NDC80 kinetochore complex component (NDC80), which was also up-regulated in both human PC and PC cell lines. Furthermore, a siRNA experiment silencing NUF2 performed in Panc-1 cells confirmed that lncRNA AF339813 was positively regulated by NUF2. Subsequent siRNA experiments demonstrated that lncRNA AF339813 induced apoptosis via mitochondria and caspase dependent pathways. Besides, lncRNA AF339813 regulated cell cycle via cyclin D1 and CDK4/CDK6 complex. It is important to clarify the exact mechanism of apoptosis related to lncRNAs in PC, knowing that it can help understand cancer invasion, metastasis, drug resistant or recurrence.

**Figure 1:**
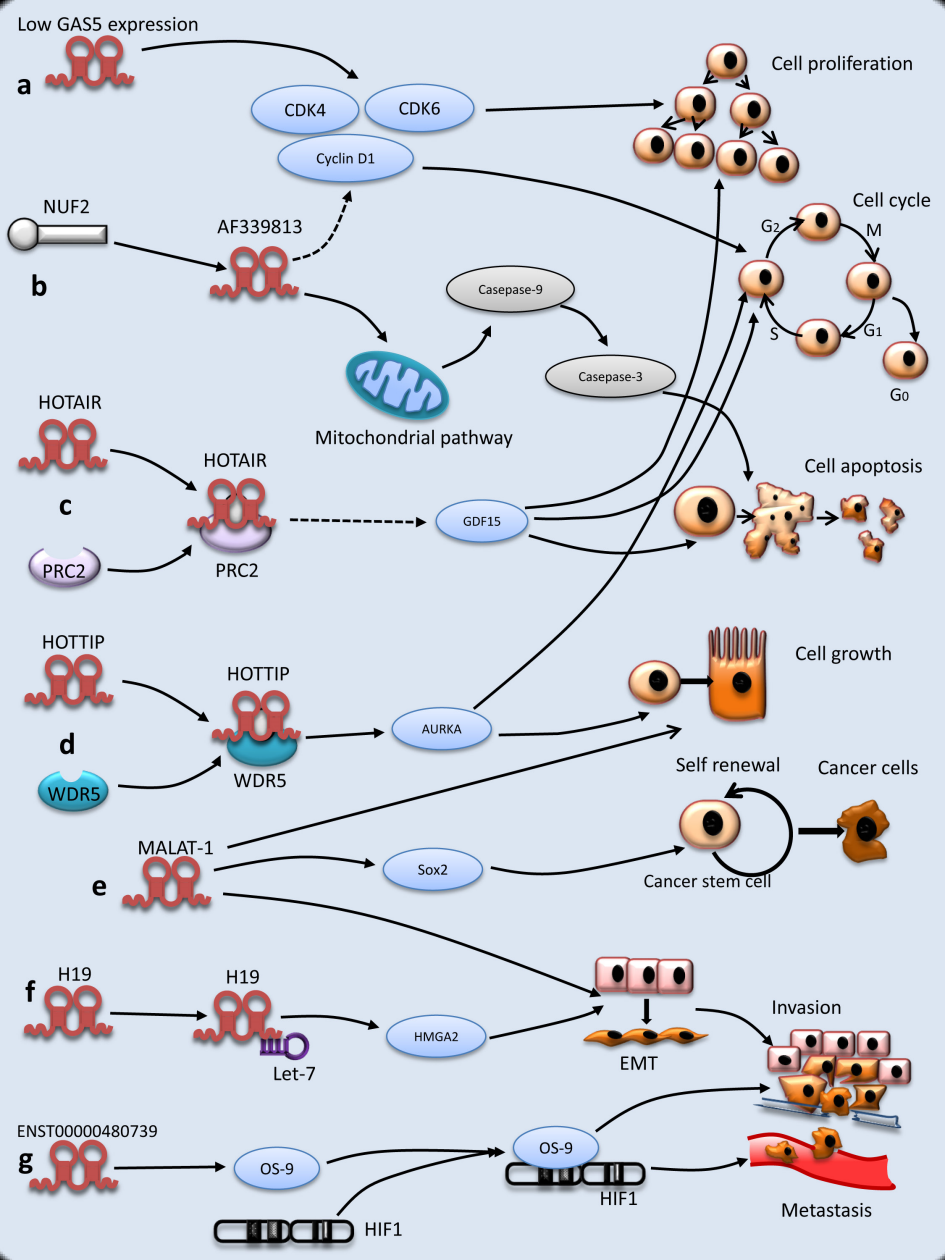
Molecular mechanisms of lncRNAs underlying PC tumorigenesis **a.** The expression of GAS5 is decreased and regulates cell proliferation. **b.** NUF2 up-regulates lncRNA AF339813. LncRNA AF 339813 regulates apoptosis and cell cycle. LncRNA AF339813 regulates apoptosis via mitochondrial and caspase pathways. LncRNA AF339813 regulates cell cycle through cyclin D_1_ and CDK4/CDK6. **c.** HOTAIR is physically combined with PRC2, which increases cell proliferation and decreases apoptosis through GDF15. **d.** Similar to HOTAIR, HOTTIP is combined with protein WDR5 and then regulates cell growth and cell cycle, but the mechanism is complex. **e.** MALAT-1 promotes EMT, PC cell growth and proliferation (not shown). Besides, MALAT-1 maintains stemness by regulating Sox2. **f.** As a molecular sponge, H19 antagonizes let-7 and increases EMT through HMGA2 pathway. **g.** ENST000000480739 facilitates PC invasion and metastasis through OS-9/HIF-1 pathway.

In summary, lncRNAs play diverse roles in the initiation and progress of PC by regulating cell proliferation and cell cycle, suppressing cell apoptosis, and promoting invasion and metastasis (Figure 1). However, there is little knowledge about lncRNAs related to PC, and therefore further investigations are needed to elucidate the molecular mechanism in PC tumorigenesis.

## POTENTIAL UTILITY OF LNCRNAs IN PC

### lncRNAs in PC diagnosis and prognosis

PC is the fourth leading cause of cancer-related death in the USA, with a 5-year-survival rate about 5% [67]. Although comprehensive therapies have been used for the treatment of PC, the therapeutic outcome of PC patients remains unsatisfactory. It is therefore urgent to identify early diagnosis biomarkers and prognostic factors related to the outcome of PC patients for the sake of choosing individualized therapy strategies for PC patients [68].

Early diagnosis and appropriate treatment can reduce the mortality of PC effectively. Several serum tumor biomarkers have been employed for detecting PC, such as carbohydrate antigen-19.9 (CA19-9), carbohydrate antigen 242 (CA242) and carcinoembryonic antigen (CEA) [69]. However, given the low sensitivity and specificity of CA19-9 in the early stage of PC, it is urgent to search novel tumor biomarkers to distinguish PC patients from health people.

An emerging understanding about the potentiality of lncRNAs as a new type of cancer biomarkers for the diagnosis, treatment and prognostic prediction has renewed our knowledge about “junk DNA”. Due to tissue-specific expression of lncRNA, it is of great importance in distinguishing different types of cancer and cancer subtypes. Some lncRNAs have been tested for clinical applications. It has been proved that the expression level of lncRNA prostate cancer antigen 3 (PCA3) in prostate cancer tissue is significantly higher than that in benign prostate tissue [70]. Currently, several studies reported that PCA3 could be used as a biomarker with high sensitivity and specificity in diagnosing prostate cancer [18, 71, 72, 73]. What is more, lncRNAs have shown great potential utilities in non-invasive detection of cancer. Zhou et al [74] selected 8 lncRNAs in plasma as candidate targets to identify suitable biomarkers for the diagnosis of gastric cancer and found that the expression level of plasma H19 in gastric cancer was significantly higher than that in the controls [74]. Besides, the plasma level of H19 was decreased markedly in postoperative samples.

H19 was considered as tumor suppressor gene in PC [75] but loss of imprinting of H19 was not related to PC carcinogenesis [76]. However, dysfunction of several other lncRNAs has been identified, which provides a possible opportunity to clarify the mechanism of PC initiation and progression. For instance, Ye et al [77] used a bioinformatics computational approach to predict lncRNA-ralated regulation mechanisms in PC on the basis of high-throughput sequencing (CLIP-Seq) data from StarBas. They found that 21 lncRNAs involved in lncRNA-miRNA-mRNA regulation were differentially expressed in PC and normal tissues, indicating lncRNAs play important roles in the transcriptional regulation during the initiation and progression of PC. The abnormal expression of lncRNAs in PC provides new cues for understanding the mechanism of tumorigenesis and shows a potential diagnosis value in PC. Müller et al [78] found that 43 lncRNAs and other RNAs including mRNA, miRNA and sdRNAs were differentially expressed in six PC and five control tissues by next-generation sequencing. Therefore, some kinds of lncRNAs may become novel biomarkers of PC. In addition, lncRNAs have seen a great potential value in the clinical diagnosis of PC. Wang et al [79] employed Arraystar Human LncRNA Microarray to screen potential biomarkers in 7419 lncRNAs of PC and found that HOTTIP-005, XLOC_006390 and RP11-567G11.1 were increased markedly in PC tissue. Besides, the derived RNA fragments of HOTTIP-005 and RP11-567G11.1 named HDRF and RDRF were increased significantly as compared with the controls. Notably, Xie et al [80] suggested that the expression of salivary HOTAIR and PVT1 could serve as potential non-invasive biomarker for detecting PC. However, the use of lncRNAs as a non-invasive examination of PC is relatively rare so far.

Several recent studies have demonstrated that some lncRNAs correlate with the clinicopathological characteristics and the prognosis of PC patients, and therefore may prove to be ideal clinical biomarkers for predicting the prognosis of PC.

As previous described, HOTAIR can serve as an “onco-lncRNA” due to its high expression in PC. It was found that HOTAIR was a negative prognostic factor related to poor OS and lymph node metastasis [33]. Ye et al [81] measured a lncRNA expression profile of PC by microarray and selected Actin filament associated protein 1 antisense RNA(AFAP1-AS1) as a prognostic marker. They found that it was associated with lymph node metastasis, perineural invasion and poor survival. The result of RT-qPCR in evaluating the expression level of AFAP1-AS1 in PC showed that the areas under receiver operating characteristic curve (ROC curve) could reach 0.8669 when AFAP1-AS1 was used as a prognostic marker of PC. Li et al [82] analyzed the expression of mRNA and lncRNA in PC tissue through microarray platform and found that 1881 lncRNAs were up-regulated, and 3369 lncRNAs were down-regulated in PC tissues as compared with non-cancerous tissues. In addition, the expression level of BC008363 might be a biomarker for predicting the prognosis of PC. Liu et al [83] detected the expression level of MALAT-1 in 45 formalin-fixed paraffin embedded (FFPE) PC and 25 FFPE adjacent tissues. Another similar study concentrating on MALAT-1 had the same conclusion [84]. These studies indicate that MALAT-1 was un-regulated during the progress of PC and could be a potential prognostic biomarker for PC patients. Surprisingly, the expression of some lncRNAs showed the opposite effect on prognosis. Peng et al [85] revealed that lncRNA HULC was over-expressed in human PC tissue compared with corresponding normal tissue. Importantly, the higher level of HULC was correlated with large tumor size, advanced lymph node metastasis and vascular invasion. Moreover, multivariate analyses suggested that HULC could serve as an independent predictor for OS of PC patients. Huang and colleagues [86] showed that the expression level of lncRNA plasmacytoma variant translocation 1 (PVT1) was increased in PC tissue compared with adjacent tissue. The expression level of PVT1 was correlated with the clinical stage and N-classification in PC. Similar to HULC, PVT1 can serve as an independent prognostic factor for poor OS in PC patients. Unlike other lncRNAs, reduced lncRNA LOC285194 was an independent poor prognostic factor closely associated with tumor progression in PC patients [55].

### lncRNAs in PC therapy

The clinical application of some PC-targeted drugs such as Gemcitabine does not seem to have improved the OS of PC patients and the effect of this therapy remains controversial. To date, some LncRNAs have been found to be associated with PC therapy. Li and colleagues made use of high-throughput microarray to detect the expression profiles of lncRNAs in PC tissues, and found that the expression of HOTTIP was up-regulated in PC by real-time PCR [87]. Importantly, they also reported that HOTTIP promoted Gemcitabine resistance through HOXA13, implying that HOTTIP and HOXA13 may be novel therapeutic targets for PC. Unfortunately, they failed to identify the downstream signaling pathway regulated by HOTTIP. Another lncRNA related to Gemcitabine was PVT1. You et al [88] found that PVT1 gene could regulate Gemcitabine sensitivity in the human ASPC-1 PC cell line. They demonstrated that Gemcitabine sensitivity was increased when full length PVT1 cDNA was over-expressed in the antisense orientation, while Gemcitabine sensitivity was decreased when full length PVT1 cDNA was over-expressed in the sense orientation. Regretfully, the mechanism of PVT1 regulating Gemcitabine underlying PC tumorigenesis remains unknown. Besides, Jiao and colleagues [53] revealed that MALAT-1 could decrease chemosensitivity of Gemcitabine in AsPC-1 and CFPAC-1 cell lines.

Recently, a phase 1/2a study of BC-819 in patients with unresectable PC was conducted in multicenters and this is a novel therapeutic strategy focusing on lncRNA H19 [89]. BC-819 is a double-stranded DNA plasmid regulating the expression of diphtheria-toxin gene controlled by H19 regulatory sequences [90]. The study demonstrated that CT- or endoscopic ultrasound-guided injection of BC-819 was safe at the applicable dose, suggesting that BC-819 injection may be a novel therapeutic strategy for the clinical treatment.

In summary, the research and application of lncRNAs as a molecular target for PC treatment are still in the infantile stage, and the function of many lncRNAs related to PC has not been fully characterized.

## PROSPECTIVE

PC ranks fourth leading cause of cancer-related death with poor prognosis in the United States [91]. Due to the lack of early diagnostic biomarkers and rapid progression, PC remains a high-mortality malignant disease. On the one hand, environmental factors such as westernized diet, tobacco and alcohol are considered as risk factors of PC [92]. On the other hand, hereditary factors including Kras mutation partially contribute to the pathogenesis of PC [93]. Currently, CA19-9 was reported to be a biomarker for the diagnosis and prognostic prediction of cancer [69, 94]. However, it is not an ideal tumor marker for early diagnosis of PC due to the relatively high false positivity and a poor positive predictive value. It is therefore an urgent task to find a novel tumor biomarker and therapeutic target for the early diagnosis and prognostic prediction of PC.

Recently, several studies have demonstrated that some LncRNAs are associated with clinicopathologic characteristics of PC and these lncRNAs may be potential tumor biomarkers or/and therapeutic targets of PC. Given the multiple biological functions of lncRNAs in epigenetic regulation, the molecular mechanism in the initiation and progression of PC related to lncRNAs is a new layer of complexity. Although some prominent functions of lncRNAs (such as HOTAIR, HOTTIP, PVT1 and GAS5) have been elucidated in previous studies, the understanding about the roles of lncRNAs in the carcinogenesis of PC is still fractional and segmentary. Currently available studies have shown that lncRNAs have effects on cell growth, proliferation, invasion and metastasis, though the details of these mechanisms need to be further clarified. There is still a long to go before we can identify, characterize and elucidate the actual functions of lncRNAs in the pathogenesis of PC at a molecular level and use them to clinical practice. In conclusion, lncRNAs are involved in the initiation and development of PC, as well as in multiple processes in epigenetic regulation of cell biology. Further study should be conducted to address the molecular mechanism underlying PC carcinogenesis.

## Abbreviations

HOTAIR: HOX transcript antisense RNA
PRC2: Polycomb Repressive Complex 2
HOTTIP: HOXA transcript at the distal tip
HOXA: homeobox A cluster
HOXB: homeobox B cluster
HOXD: homeobox D cluster
MALAT-1: metastasis associated in lung adenocarcinoma transcript 1
Sox2: SRY (sex determining region Y)-box 2
OS-9: osteosarcoma amplified-9
AFAP1-AS1: Actin filament associated protein 1 antisense RNA
PVT1: plasmacytoma variant translocation 1
GAS5: growth arrest-specific 5
CDK6: cyclin-dependent kinase 6
CDK4: cyclin-dependent kinase 4
PC: Pancreatic cancer
NUF2: Ndc80 kinetochore complex component
GDF15: growth differentiation factor 15
WDR2: WD repeat containing protein 2
EMT: epithelial-mesenchymal transition
HMGA2: High Mobility Group-A2
HIF-1: hypoxia-inducible factor 1.
